# Editorial: Schema modes, case conceptualization, and psychotherapy process: qualitative explorations of clinical practice

**DOI:** 10.3389/fpsyt.2024.1377069

**Published:** 2024-02-21

**Authors:** David Edwards, Eshkol Rafaeli, Pam Pilkington

**Affiliations:** ^1^ Department of Psychology, Rhodes University, Makhanda, South Africa; ^2^ Psychology Department and Gonda Multidisciplinary Brain Research Center, Bar-Ilan University, Ramat Gan, Israel; ^3^ Faculty of Health Sciences, Australian Catholic University, Melbourne, VIC, Australia

**Keywords:** schema therapy, qualitative research, case based research, clinical practice, schema modes

Many clinical problems have their source in experiences of overwhelming emotion, often from early in life, when young children do not have the resources to handle experiences of intense distress resulting from adversity, unmet needs, and trauma. The resulting implicit patterns, which schema therapists call Early Maladaptive Schemas, are often complex and can be understood as structures and sequences of specific schema modes. Lazarus and Rafaeli (1) define these as “identifiable units characterized by specific combinations of affect, behaviors, cognitions, and desires … that tend to be co-activated in a meaningful and lawful manner for limited periods of time.”

Although much of the schema therapy literature focuses on only a small number of such modes, in a review of modes relevant to clinical practice, Edwards differentiated over 80 modes. He also identified important mode processes such as blending, sequencing, and flipping. Most of these have been recognized and described by researchers who are also clinicians facing the challenges of conceptualizing their own cases.

For this Research Topic, we sought clinically-focused articles that highlighted the subtleties of the mode processes seen in clinical practice. Given that psychotherapy is an applied science, such clinically focused explorations are an essential part of the scientific process. As Flyvbjerg (2) asserts:

“… a scientific discipline without a large number of thoroughly executed case studies is a discipline without systematic production of exemplars, and a discipline without exemplars is an ineffective one.”

This applies to the systematic use of case examples and case studies to instantiate theory and link it to practice as well as to other qualitative explorations that engage directly with the experiences of therapists and clients.

This Research Topic, which includes Edwards’ article, features two case studies. Bachrach et al.’s case of the schema therapy treatment of dissociative identity disorder provides a lucid example of the complexity of the treatment and the role of identifying modes in developing a case conceptualization that can guide the treatment. The case material is relevant to many significant questions that arise in the treatment of such complex cases, and the fascinating description of the process of gradually reducing the impact of Ella’s punitive mode and eventually shrinking it and chopping it into pieces bears on the perennial debate with respect to whether parts of the self, rather than being integrated, can be dissolved or banished. Their account points to some of the common inconsistencies in the schema therapy literature in regard to differentiating and naming modes. For example, although they identified 17 different “parts” in the client, Ella, five of these were categorized under the same broad mode – Detached Protector. Furthermore, some of these seem to have strong elements of overcompensation.

Boog and Tibboel’s hybrid case example highlights ways of responding to the challenges posed by the automaticity of mode processes, with specific reference to addiction. They examine how automaticity has been conceptualized as having several components and highlight the role of the Healthy Adult in developing meta-awareness or mindfulness to enable the interruption of highly automatized mode processes. The case shows how this can develop over time and the clinical problems that must be faced in the process.

Rather than focus on a single case, Simpson and Navot provide a range of case vignettes to illustrate the important distinction between genuine vulnerability in the client and several modes that exhibit pseudo-vulnerability. With clients in these modes, the therapist feels pressure to offer care and assistance, but these clients are not in a state to accept real therapeutic help. Rather, they want to be rescued or complain and show others how hard done by they are, without doing anything about it. The authors show how this problem has long been recognized within many approaches to psychotherapy and how the schema therapy model offers practical means to address it both in terms of case conceptualization and specific interventions.

Three of the articles are based on semi-structured interviews exploring specific clinical problems. Yakin and Arntz focus on the mode processes involved in teaching the client to relate to Vulnerable Child states in an accepting and healing manner. Relating adaptively to the Vulnerable Child modes is central to positive outcomes in schema therapy. Participants who evidenced a well-developed capacity to do this were invited to complete structured interviews. The interviews included the use of Bernstein’s iModes cards to bring the experience of the Vulnerable Child modes to life. The material allowed participants to identify specific aspects salient to the process that can serve as a helpful guide to clinicians as they encourage clients to develop and strengthen these capacities.

The focus of the article by Semeniuc et al. is the experience of therapists facing the challenge of keeping in their Healthy Adult mode when faced with the avoidant and over-compensatory behaviours of clients with obsessive-compulsive disorder. Participants were guided to vividly imagine such interactions and track their experiences as they unfolded. This method allowed the researchers to identify a range of mode processes and to differentiate those involved when therapists were able to maintain a Healthy Adult stance from those when they could not.

Josek et al. used semi-structured interviews to get a detailed understanding of the range of different client responses to engaging in chairwork. Chairwork is an important means of helping clients recognize their different modes, recognize how they relate to each other, and work towards resolving conflicts between them. Their thematic analysis points to areas where therapists might learn to be more aware of the difficulties some clients face when invited to engage in chairwork. It also highlights aspects of the positive therapeutic impact of chairwork that many clients experience.

These articles all draw on qualitative data to provide a trustworthy basis for guiding clinical action. They highlight important aspects of the clinical realities found in a range of different contexts in a manner that will be of direct value to clinicians.

## Author contributions

DE: Writing – original draft, Writing – review & editing. ER: Writing – review & editing. PP: Writing – review & editing.
